# Post-traumatic growth trajectories and influencing factors in patients undergoing hip and knee arthroplasty: a prospective longitudinal study

**DOI:** 10.3389/fpsyt.2025.1556829

**Published:** 2025-08-04

**Authors:** Yan Xie, Zhongmin Fu, Peifang Li, Jiali Chen, Lin Zhang, Ying Liu, Ning Ning, Zongke Zhou

**Affiliations:** ^1^ West China School of Public Health and West China Fourth Hospital, Sichuan University, Chengdu, China; ^2^ Department of Neurosurgery, Affiliated Hospital of Zunyi Medical University, Zunyi, China; ^3^ Department of Orthopedics, West China Hospital, Sichuan University, Chengdu, China

**Keywords:** hip arthroplasty, knee arthroplasty, post-traumatic growth, influencing factors, social support, self-efficacy, coping strategy

## Abstract

**Background:**

Total hip/knee arthroplasty (THA/TKA) has emerged as the gold-standard treatment for end-stage osteoarthritis. However, persistent postoperative pain and surgical trauma often induce psychological distress, complications that are frequently overlooked despite high procedural success rates. Leveraging Post-Traumatic Growth (PTG) theory, this study longitudinally investigated PTG levels. Through multidimensional analysis of predictive factors, we can provide an evidence-based framework for psychological interventions to accelerate rehabilitation.

**Methods:**

This prospective longitudinal study included 160 patients undergoing joint replacement surgery for osteoarthritis (79 hip and 81 knee patients). In addition to collecting demographic and disease-related data, several assessments were conducted at four time points: preoperative day 1 (T1), postoperative day 1 (T2), postoperative month 1 (T3), and postoperative month 3 (T4). The instruments used included the Post-Traumatic Growth Inventory (PTGI), General Self-Efficacy Scale (GSES), Perceived Social Support Scale (PSSS), Medical Coping Modes Questionnaire (MCMQ), Harris Hip Score (HHS), and Knee Society Score (KSS). Univariate analysis and stratified regression were applied to explore the influencing factors.

**Results:**

PTG scores showed a statistically significant nonlinear progression characterized by: preoperative day 1 (27.23 ± 12.87), postoperative day 1 (48.61 ± 14.49), postoperative month 1 (42.87 ± 12.72), and postoperative month 3 (64.37 ± 9.42). Housing type (T1, T2, T4), arthritis location (T1), surgical site (T2), complications (T4), comorbidities (T1), and joint function (T1–T4) were significant predictors of PTG (P < 0.05). Self-efficacy was positively correlated with PTG at T1 and T2 (P < 0.05). Coping strategies, such as “facing” (T1, T4), were positively correlated with PTG, while “avoidance” (T1, T2, T4) and “yielding” (T1) showed negative correlations (P < 0.05). Family support (T2, T3) was positively correlated with PTG (P < 0.05).

**Conclusions:**

The PTG scores in THA/TKA patients demonstrated a curvilinear upward trajectory over time, maintaining moderate to low overall levels. Notably, the 1-month postoperative period emerged as a critical window for targeted interventions. Joint function, self-efficacy, proactive coping strategies, and social support significantly enhanced PTG levels, whereas yield-avoidant coping strategies showed negative correlations with PTG scores. Healthcare providers should monitor multidimensional factors influencing PTG variations across different time points, formulating evidence-based health education programs and personalized interventions to accelerate rehabilitation processes.

## Introduction

Osteoarthritis (OA) is a common chronic joint disease characterized by degenerative changes in articular cartilage and bone hypertrophy, leading to joint swelling, pain, and functional impairment. Over 500 million people worldwide are affected by OA (1–3). Total hip arthroplasty (THA) and total knee arthroplasty (TKA) have become the preferred treatment options for end-stage OA (3). However, 44.4% of patients scheduled for THA/TKA experience negative emotions prior to surgery, which are often overlooked. During postoperative rehabilitation, issues such as pain and joint maladaptation frequently arise, and more than 20% of TKA patients report dissatisfaction with surgical outcomes. The conventional biomechanical evaluation framework fails to adequately account for this gap in patient satisfaction (4–6).

The theory of post-traumatic growth (PTG) highlights a four-phase pathway—disruption of core beliefs, cognitive-emotional processing, resource regulation, and positive transformation—to explain the psychological mechanisms through which individuals experience positive changes following trauma (7, 8). PTG has been shown to be positively associated with quality of life and rehabilitation adherence in various medical stress contexts, including major surgery, cancer, accidents, and chronic illness (7, 9). In the orthopedic field, relevant studies suggest that PTG contributes to an expanded understanding of life among patients with rheumatoid osteoarthritis and offers practical guidance for promoting health (10). Furthermore, PTG levels have been positively correlated with concurrent quality of life in patients with hip fractures (11). In patients with OA, the chronic pain associated with the disease and the traumatic stress of joint replacement surgery together constitute significant stressors, meeting the PTG model’s criteria for core belief disruption through both prolonged and acute stressors (12–14). During the postoperative rehabilitation process, THA/TKA patients face persistent psychological stress due to pain, discomfort, and unmet high expectations (4, 15). Factors such as self-efficacy and social support facilitate cognitive restructuring and resource regulation (16, 17), theoretically forming a closed-loop pathway from distress and rumination to eventual growth.

For joint diseases, PTG in patients with rheumatoid arthritis enhanced their knowledge of living with the disease and provided practical health-promoting advice (10). Progressive OA leads to chronic pain, functional impairment, surgical trauma, and postoperative rehabilitation discomfort, all of which may trigger anxiety and depression across disease stages (18–20). However, systematic investigations into PTG and its determinants in THA/TKA patients remain scarce, resulting in clinical interventions predominantly focused on functional rehabilitation while lacking evidence-based strategies for psychological growth (6, 21, 22).

This study therefore employs PTG theory to identify modifiable predictors—including coping styles, self-efficacy, and social support—to establish an evidence-based framework for targeted psychological interventions, ultimately enhancing functional recovery and subjective well-being.

## Methods

### Study design

This study is a prospective longitudinal study conducted using a convenience sampling method. After admission, trained research team members explained the study’s purpose and procedures to the patients and invited them to participate. The study was conducted following the principles of the Declaration of Helsinki and the Strengthening the Reporting of Observational Studies in Epidemiology (STROBE )guidelines, and it was approved by the Ethics Committee of West China Hospital, Sichuan University (Approval No. 1349). All participants signed an informed consent form prior to their involvement in the study. All patients completed a 3-month postoperative follow-up.

### Participants

This study was conducted from September 2022 to November 2023 in the Department of Orthopedics at West China Hospital, Sichuan University. A total of 163 patients diagnosed with OA participated in the study, including 81 patients undergoing THA and 82 patients undergoing total knee arthroplasty TKA. The inclusion criteria were as follows: (1) age ≥ 18 years; (2) diagnosis of primary hip or knee OA; (3) undergoing primary unilateral total hip or knee arthroplasty; (4) basic literacy skills and the ability to communicate effectively. Exclusion criteria included patients with severe heart, liver, or kidney dysfunction, malignancy, or those who had experienced other significant events within the past year.

### Sample size calculation

The sample size was calculated using PASS 15 software. PTG scores were used as the primary outcome variable. Based on preliminary survey results, the standard deviation was 23, and the allowable error was 4. Using a two-sided test (α = 0.05, β = 0.8), the required sample size was calculated as N = 130. Considering a 20% invalid questionnaire rate, the final required sample size was 163 participants.

### Data collection

To ensure the quality of the surveys, the research instruments were assessed and reviewed by an orthopedic expert team, and the researchers were strictly trained before conducting the data collection. The research instruments included:

①Posttraumatic Growth Inventory (PTGI): The total scale had a Cronbach’s α coefficient of 0.874. For its five dimensions (Personal Strength, New Possibilities, Spiritual Change, Relating to Others, and Appreciation of Life), the Cronbach’s α coefficients ranged from 0.611 to 0.796 (23).
②Medical Coping Modes Questionnaire (MCMQ): This scale includes three dimensions—Confrontation, Avoidance, and Resignation—with Cronbach’s α coefficients of 0.64, 0.85, and 0.67, respectively (24).
③The General Self-Efficacy Scale (GSES) is a unidimensional instrument comprising 10 items, rated on a 4-point Likert scale (1–4). The total score ranges from 10 to 40, with higher scores indicating greater self-efficacy. The scale demonstrated good internal consistency, with a Cronbach’s alpha coefficient of 0.87 (25).
④Perceived Social Support Scale (PSSS): The Cronbach’s α coefficients for the overall scale and its three dimensions (Family Support, Friend Support, and Other Support) ranged from 0.813 to 0.840 (26).
⑤Harris Hip Score (HHS): This scale assesses hip joint function, including pain, function, deformity, and range of motion, with a maximum score of 100. Scores are categorized into four levels: excellent (>90), good (80–89), fair (70–79), and poor (<70) (27).
⑥Hospital for Special Surgery Knee Score (HSS): This scale evaluates knee joint function, including six aspects: pain, function, range of motion, muscle strength, flexion deformity, and stability. The total score is 100, categorized into four levels: excellent (>85), good (70–84), fair (60–69), and poor (≤59) (28).

The PTGI, MCMQ, GSES, and PSSS were used to assess PTG (higher scores indicate higher PTG levels), coping strategies (higher scores indicate a stronger tendency toward specific coping strategies), self-efficacy (higher scores indicate a higher sense of self-efficacy), and social support (higher scores indicate higher social support), respectively. Joint function was assessed using the Harris Hip Score (HHS) (27) or Knee Society Score (KSS) (28).

Demographic data collected included sex, age, ethnicity, body mass index (BMI), marital status, education level, occupation, long-term residence, housing types, living arrangements, family income, payment method, and exercise. Disease-related data included arthritis location, disease duration, comorbidities, albumin levels at admission, surgical site, smoking and drinking history, past medical history, blood transfusion (during hospitalization), previous hospitalizations, and postoperative complications.

Demographic data, disease-related data, and the MCMQ were collected on preoperative day 1 (T1); PTGI, GSES, PSSS, and HHS/KSS were collected on T1, postoperative day 1 (T2), postoperative month 1 (T3), and postoperative month 3 (T4). Data from T1 and T2 were collected in the ward, and data from T3 and T4 were collected via telephone follow-up or outpatient visits. T1 was considered the baseline psychological state after prolonged suffering from osteoarthritis, with its score reflecting the potential for growth under the cumulative trauma of chronic illness rather than an immediate reaction to the acute surgical event. Postoperative time points (T2–T4) were directly associated with the surgical experience. Since the majority of pain relief and significant improvements in daily functional ability after THA/TKA occur within 1 to 3 months postoperatively, focusing on this critical rehabilitation period aimed to capture key turning points in the dynamic trajectory of PTG, thereby providing a crucial window of opportunity for early intervention.

### Statistical analysis

For continuous data, means and standard deviations were used for normally distributed data, while medians and interquartile ranges were used for non-normally distributed data. Categorical data were expressed as frequencies and percentages. Univariate analyses were performed using the Mann-Whitney U test, independent t-test, one-way analysis of variance, repeated measures analysis of variance, and Spearman correlation analysis. Multivariate analysis was conducted using stratified regression to explore the influence of medical coping strategies, self-efficacy, and social support on PTG scores, after controlling for demographic and disease-related factors. All data analyses were performed using SPSS 26.0 software, with a two-sided significance level of α = 0.05.

## Results

All patients completed follow-up at four time points. Initially, 163 patients met the inclusion criteria. However, one patient undergoing TKA was lost to follow-up due to a change in contact details, and two patients undergoing THA withdrew from the study. As expected, there were no statistically significant differences in baseline data between the 160 patients (P > 0.05). The baseline characteristics of the 160 patients are shown in **Table 1**.

**Table 1:** General information of patients (N=160).

Variable	Category	Number	Percentage (%)
Age (years)	≤40	7	4.4
	41~50	10	6.2
	51~60	56	35
	61~70	44	27.5
	≥70	43	26.9
Sex	Male	34	21.2
	Female	126	78.8
Ethnicity	Ethnic Minority	13	8.1
	Han	147	91.9
BMI	Underweight	4	2.5
	Normal	64	40.6
	Overweight	30	18.8
	Obese	62	38.1
Education Level	Elementary or below	75	42.5
	Middle School	44	28.7
	College/Undergraduate	19	13.8
	High School	19	13.1
	Graduate and above	3	1.9
Marital Status	Divorced	6	3.8
	Widowed	19	11.9
	Unmarried	2	1.3
	Married	133	83.1
Occupation	Mental Labor	23	15
	Physical Labor	53	26.3
	Retired	45	31.9
	Unemployed	39	26.8
Long-Term Residence	Urban	101	63.1
	Rural	59	36.9
Housing Type	Elevator House/Flat	96	60
	Stairs House	64	40
Living Arrangement	Living Alone	36	6.9
	Living with Parents	3	1.9
	Living with Spouse	98	73.1
	Living with Children	23	18.1
Monthly Family Income (Yuan)	<3000	5	3.1
	3000-4999	23	14.4
	5000-10000	80	50
	>10000	52	32.5
Payment Method	Insurance Payment	139	85
	Out-of-pocket	21	15
Disease Duration (years)	<5	52	32.5
	6-10	53	33.1
	11-19	24	15
	≥20	31	19.4
Exercise	None	117	73.1
	Anaerobic Exercise	2	1.3
	Aerobic Exercise	41	25.6
Smoking History	None	149	93.1
	Yes	11	6.9
Drinking History	None	152	95
	Yes	8	5
Past Medical History	None	65	40.6
	Yes	95	59.4
Comorbidities	None	73	45.6
	Yes	87	54.4
Serum Albumin Level at Admission	Normal	158	98.8
	Low	2	1.3
Surgical Site	Hip Joint	79	49.4
	Knee Joint	81	50.6
Postoperative Complications	None	153	95.6
	Yes	7	4.4
Blood Transfusion (during hospitalization)	No	158	98.8
	Yes	2	1.2
Arthritis Location	Unilateral	79	49.4
	Bilateral	81	50.6
Joint Function	T1: Good	24	15
	T1: Fair	21	13.1
	T1: Poor	115	71.9
	T2: Excellent	2	1.3
	T2: Good	9	5.6
	T2: Fair	21	13.1
	T2: Poor	128	80
	T3: Excellent	9	5.6
	T3: Good	34	21.3
	T3: Fair	43	26.9
	T3: Poor	74	46.2
	T4: Excellent	65	40.6
	T4: Good	51	31.9
	T4: Fair	43	26.9
	T4: Poor	1	0.6

### PTG trajectory analysis

**Figure 1:**
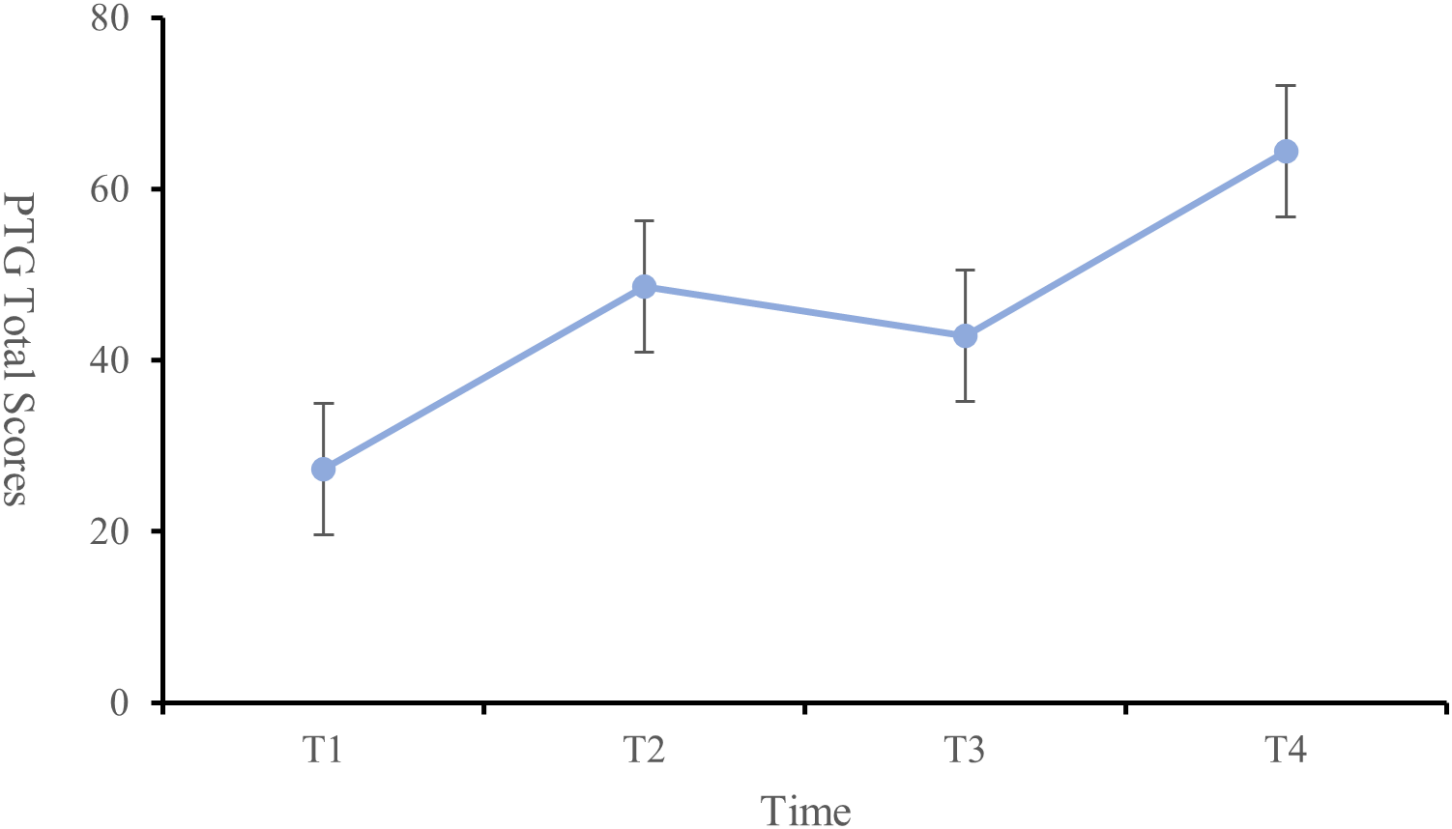
Change trend of PTG total score from T1 to T4.

**Figure 2:**
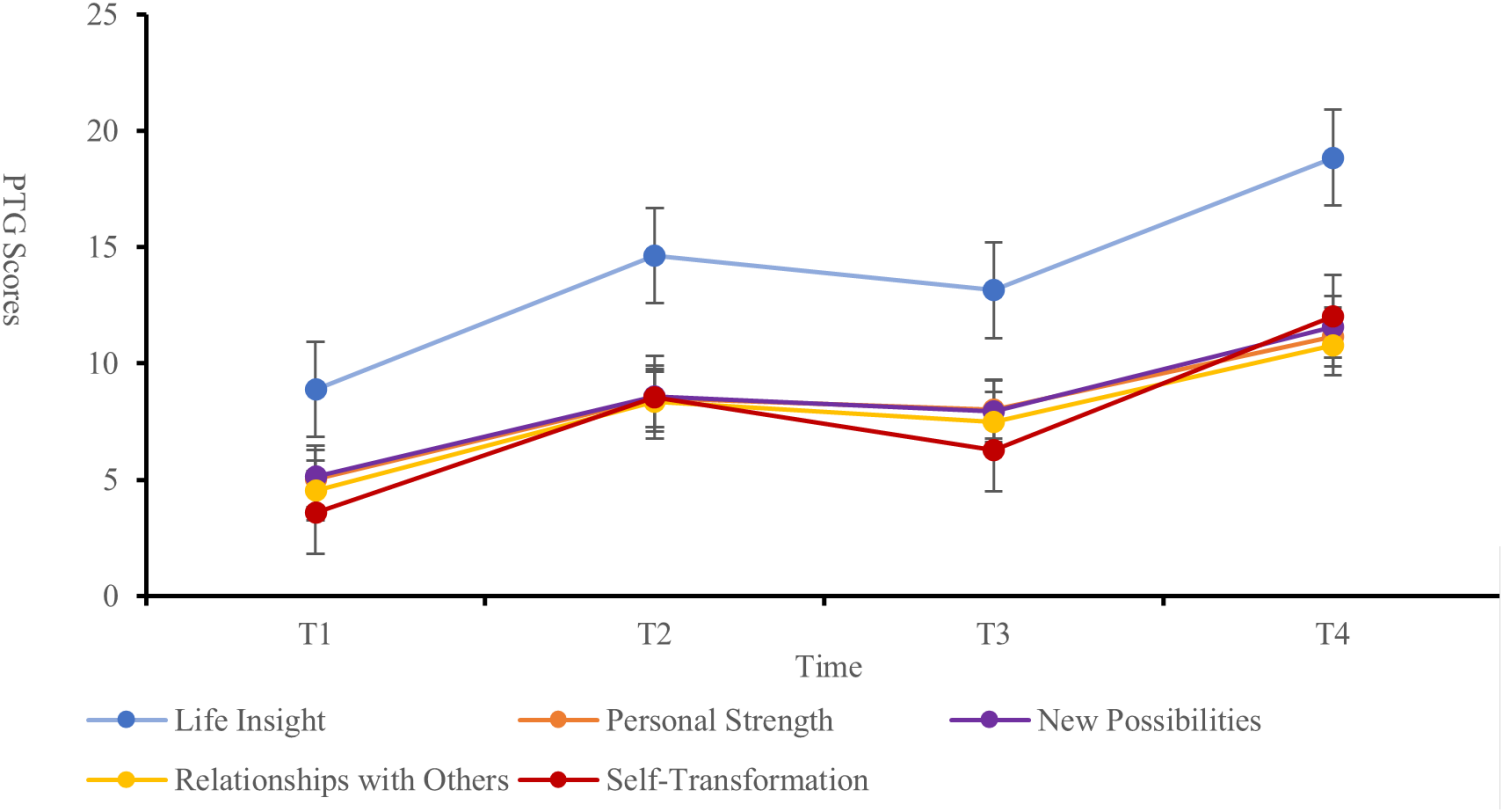
Variation trend of PTG scores in each dimension from T1 to T4.

The PTG scores of the patients at each time point were as follows: T1 (27.23 ± 12.87), T2 (48.61 ± 14.49), T3 (42.87 ± 12.72), and T4 (64.37 ± 9.42), indicating an overall moderate to low level of PTG. The changes in PTG scores across dimensions and the total score showed a trend of increasing, then decreasing, and then increasing again (**Figure 1**). Significant differences were observed in the self-transformation, life realization dimensions, and total PTG score across the four time points (P < 0.001) (**Table 2**, **Figure 2**).

**Table 2:** Longitudinal analysis of PTG dimensions and total scores at T1 to T4 time points (N=160, $\bar{x}$ ±s).

Time Point	Life Insight	Personal Strength	New Possibilities	Relationships with Others	Self-Transformation	Total Score
T1	8.89±4.34	5.01±2.67	5.13±3.05	4.55±2.25	3.59±2.94	27.23±12.87
T2	14.63±4.46	8.50±2.66	8.57±3.09	8.37±3.65	8.54±3.78	48.61±14.49
T3	13.14±2.46	8.03±2.81	7.93±3.09	7.48±2.80	6.28±3.43	42.87±12.72
T4	18.84±3.53	11.14±1.91	11.57±2.33	10.78±2.72	12.02±3.53	64.37±9.42
*F*	321.492	242.510	212.720	196.640	328.960	534.000
*P* Value	<0.001	<0.001	<0.001	<0.001	<0.001	<0.001
Pairwise Comparison	a*, b*, c*, d*, e*, f*	a*, b*, c*, e*, f*	a*, b*, c*, d*, e*, f*	a*, b*, c*, e*, f*	a*, b*, c*, e*, f*	a*, b*, c*, d*, e*, f*

a represents the comparison of scores between T1 and T2 time points;

b represents the comparison of scores between T1 and T3 time points;

c represents the comparison of scores between T1 and T4 time points;

d represents the comparison of scores between T2 and T3 time points;

e represents the comparison of scores between T2 and T4 time points;

f represents the comparison of scores between T3 and T4 time points.

“*”indicates statistically significant differences at the 0.05 (two-sided) level.

### Analysis of coping strategies, GSES, and PSSS

**Figure 3:**
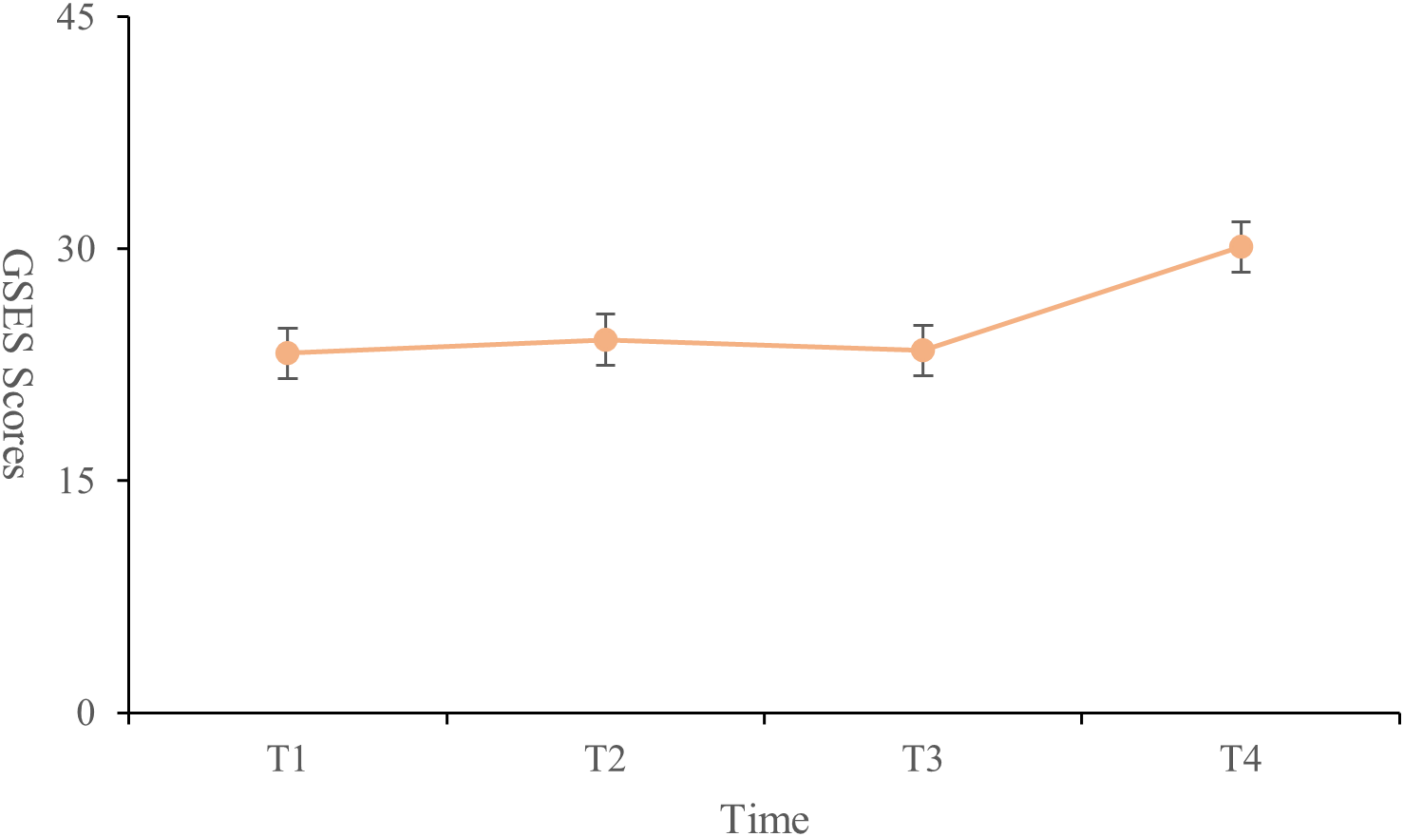
Change trend of general self-efficacy scores from T1 to T4.

**Figure 4:**
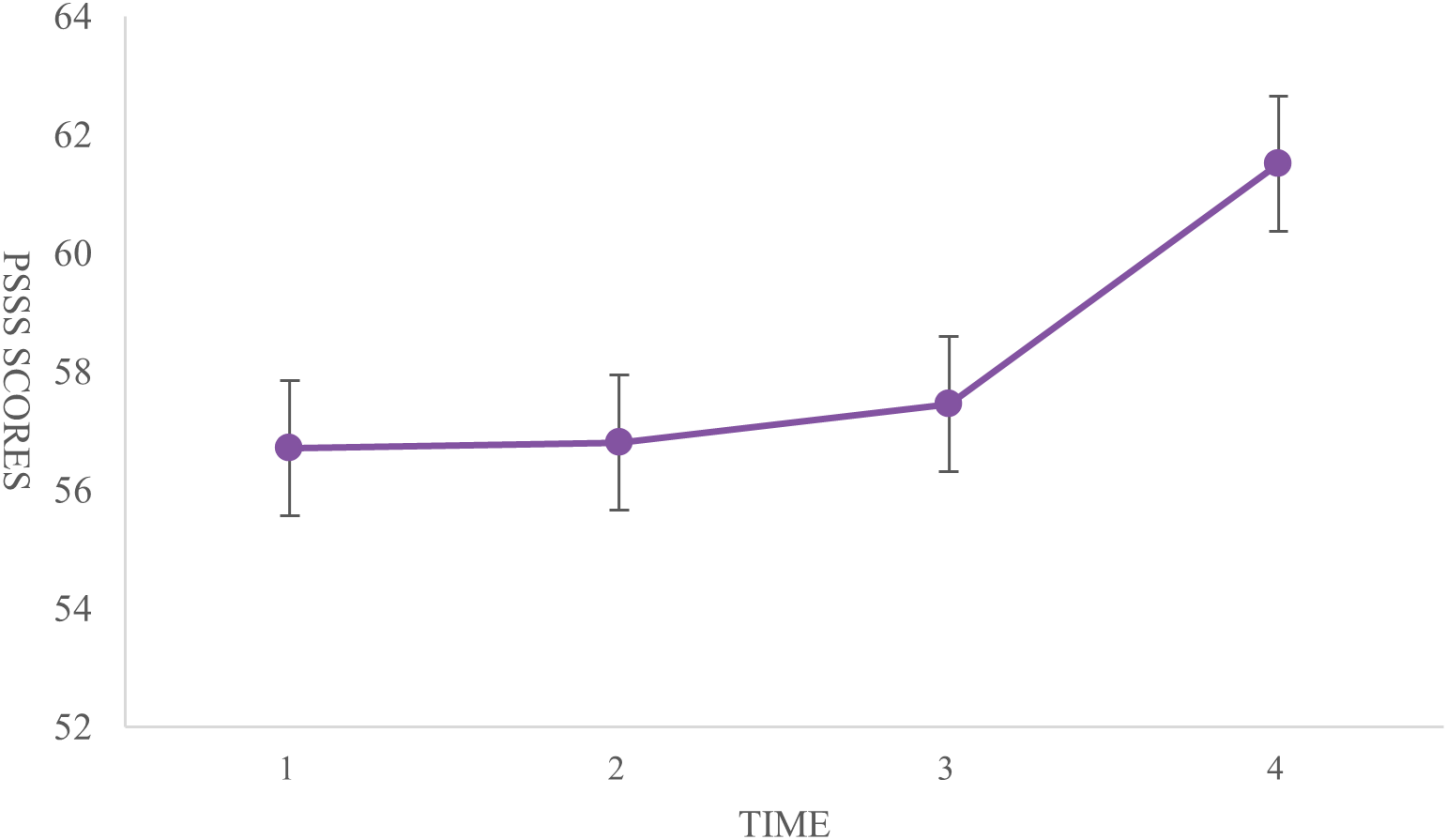
Trends in dimensions and total scores of social support from T1 to T4.

The highest score for medical coping strategies was in the avoidance dimension (18.38 ± 3.14), followed by facing (17.73 ± 3.04) and yielding (10.11 ± 2.13). General self-efficacy scores were lowest at T1 (23.24 ± 5.71) and highest at T4 (30.13 ± 4.36). The scores at T2 (24.13 ± 4.87) were higher than those at T3 (23.43 ± 4.34). Significant differences were found between T1 and T2, T1 and T4, and T2 and T4 (P < 0.001) (**Figure 3**). The total social support score showed significant differences between T1 and T4, and between T2 and T4 (P < 0.001). Significant differences were observed in the family support dimension across all four time points (P < 0.001). The friend support dimension showed significant differences only between T2 and T4 (P < 0.001). Differences in other support dimensions at T4 were statistically significant when compared to the other time points (P < 0.05) (**Table 3**, **Figure 4**).

**Table 3:** Longitudinal changes in social support dimensions and total dcores at T1 to T4 (n=160, points, $\bar{x}$ ±s).

Time Point	Family Support	Friend Support	Other Support	Total Score
T1	22.14±4.55	16.58±4.05	17.95±4.13	56.71±11.40
T2	23.12±3.51	15.89±2.44	17.79±2.30	56.80±6.15
T3	23.62±2.95	16.54±3.14	17.91±3.05	57.46±4.47
T4	25.49±2.01	17.00±2.80	19.02±2.28	61.51±5.54
*F*	39.296	5.523	8.355	28.554
*P* value	<0.001	<0.001	<0.001	<0.001
Pairwise Comparison	a*, b*, c*, d*, e*	d*	c*, d*, e*	c*, d*

a represents the comparison of scores between T1 and T2 time points;

b represents the comparison of scores between T1 and T3 time points;

c represents the comparison of scores between T1 and T4 time points;

d represents the comparison of scores between T2 and T4 time points;

e represents the comparison of scores between T3 and T4 time points.

“*”indicates statistically significant differences at the 0.05 (two-sided) level.

### Stratified analysis

Compared with living alone, patients living with a spouse at T1 (P < 0.001) or with children at T1 (P < 0.05) had lower PTG scores. At T2, patients living with a spouse had higher PTG scores (P < 0.05). At T4, patients living with children had higher PTG scores (P < 0.05) (**Table 4**). The arthritis location, comorbidities, and joint function at T1 influenced PTG levels (P < 0.05). surgical site and joint function influenced PTG levels at T2 (P < 0.05), while joint function influenced PTG levels at T3. At T4, joint function and comorbidities influenced PTG levels (P < 0.05; **Table 4**).

**Table 4:** Influencing factors of PTG level at different time points (N=160).

Variable	T1 (β)		T2 (β)		T3 (β)		T4 (β)	
	Model 1	Model 2	Model 1	Model 2	Model 1	Model 2	Model 1	Model 2
Living with Parents	0.041	-0.008	0.008	-0.044	—	—	-0.075	-0.103
Living with Spouse	-0.156**	-0.170***	0.115*	0.101*	—	—	-0.039	0.04
Living with Children	-0.083	-0.096*	0.048	0.073	—	—	0.07	0.150*
Blood Transfusion	—	—	—	—	-0.140*	-0.099	-0.097	-0.058
Payment Method	-0.084	-0.032	—	—	—	—	—	—
Comorbidities	-0.310***	-0.113**	-0.148**	-0.035	-0.209**	-0.115	-0.207**	-0.087
Number of Joint Diseases	-0.258***	-0.131***	-0.083	-0.016	-0.095	-0.06	-0.117	-0.054
Joint Replacement Site	—	—	-0.130**	-0.099*	-0.146*	-0.125*	-0.077	-0.064
Complications	—	—	—	—	-0.122	-0.118	-0.108	-0.130*
Joint Function	0.457***	0.254***	0.700***	0.566***	0.461***	0.367***	0.521***	0.451***
Exercise Habit	0.059	0.017	—	—	0.092	0.042	—	—
Self-Efficacy	/	0.267***	/	0.140***	/	0.086	/	0.061
Facing	/	0.131***	/	0.007	/	-0.017	/	0.172**
Avoidance	/	-0.144***	/	-0.158**	/	-0.114	/	-0.185**
Submission	/	-0.107**	—	—	/	-0.116	/	-0.031
Family Support	/	0.013	/	0.230***	/	0.172**	—	—
Friend Support	/	0.053	—	—	—	—	—	—
Other Support	/	0.053	/	0.026	—	—	—	—
R-Squared	0.749	0.889	0.692	0.783	0.439	0.502	0.593	0.663
△R-Squared	0.749	0.14	0.692	0.09	0.439	0.063	0.593	0.07
*F*	56.306***	77.068***	48.851***	44.094***	16.986***	12.327***	24.268***	22.096***

*P<0.05; **P<0.01; ***P<0.001; "—" indicates variables where univariate analysis at the corresponding time point showed P > 0.05; "/" indicates variables not included in Model 1.

Self-efficacy was positively correlated with PTG scores at both T1 and T2 (P < 0.001; **Table 4**). For coping strategies, in the facing dimension, scores at T1 (P < 0.001) and T4 (P < 0.01) were positively correlated with PTG scores. In the avoidance dimension, T1, T2, and T4 scores were negatively correlated with PTG scores (P < 0.01). The yielding dimension score at T1 was negatively correlated with PTG scores (P < 0.01; **Table 4**). For social support, in the social support domain, family support at T2 and T3 was positively correlated with PTG scores (P < 0.05; **Table 4**).

## Discussion

This study revealed a curvilinear trajectory of PTG among patients undergoing THA or TKA, characterized by a sharp increase immediately after surgery, a decline at one month postoperatively, and a subsequent rise at three months. Overall, PTG levels remained moderate to low. The initial surge in PTG immediately post-surgery may be attributed to effective pain management and intensive care, which activate patients’ acute positive reappraisal responses; this sense of growth often precedes actual functional recovery. At one month, the combination of intensified rehabilitation efforts and persistent residual pain may result in a gap between expectations and reality, while the reduction in medical and social support resources contributes to a decline in PTG, reflecting cognitive instability during psychological restructuring. By three months postoperatively, with improved joint function and enhanced self-efficacy, patients are more likely to complete the reconstruction of trauma-related meaning and establish a more stable and internalized form of positive psychological adaptation. This trend aligns with the curvilinear development pattern proposed in recent PTG research, emphasizing that PTG is not a linear accumulation but a dynamic process involving emotional peaks, cognitive restructuring, and adaptive integration (29, 30). These trajectory characteristics deepen our understanding of the psychological recovery mechanisms in THA/TKA patients and suggest that the first postoperative month may serve as a critical window for interventions aimed at transitioning PTG from short-term adaptation to long-term growth. Strengthening social support and external guidance for cognitive reframing during this period may enhance the stability and clinical significance of PTG.

In this study, social support and living arrangements significantly influenced patients’ PTG scores, with living arrangements showing varied effects at different time points. Although the PSSS measures relatively stable perceived social support, the event of joint replacement may prompt patients to reassess their support systems. At postoperative time points (T2–T4), surgery as a stressor may lead to renewed evaluation of the support network. At T2 (postoperative day 1), when pain peaks, perceived family support may be reinforced through caregiving behaviors. This study found statistically significant differences in perceived family support across all four time points, while friend support showed significant differences only between T2 and T4, suggesting differentiated roles of support sources during rehabilitation. These findings highlight the importance of stage-specific psychological interventions. Previous research has found that among patients living alone after total joint arthroplasty, the 90-day readmission rate was only 2.1%, showing no significant difference compared to those living with others (31). For hip fracture patients, longitudinal studies revealed that functional support (e.g., assistance with daily care) was more effective in promoting walking recovery than structural support (e.g., number of cohabitants) (31). Increased perceived levels of social support were associated with reduced stress responses to surgery and rehabilitation exercises in THA/TKA patients, leading to better adherence to recovery programs. Therefore, for patients discharged after THA/TKA, the key consideration may be whether there is a supportive environment for functional recovery, rather than living arrangements alone. When developing rehabilitation plans for patients with different living arrangements, it is recommended to comprehensively consider the degree of family support, accessibility of social resources, and the patient’s baseline functional status (31). For example, wearable remote monitoring devices and AI-assisted training have been shown to be as effective as clinical supervision (32). For patients living alone, the safety of home rehabilitation depends on preoperative functional status and appropriate modifications to the home environment, such as installing handrails or adjusting furniture height to reduce fall risk (31, 32).

Patients with comorbidities are often associated with more severe symptoms of disease (33, 34). In this study, the main comorbidities included hypertension (67.8%), diabetes (17.2%), and insomnia (13.7%). Patients with comorbidities at T1 had lower PTG levels. In surgical patients, clinically significant depressive symptoms were independently correlated with increased blood pressure when patients were aware of their hypertension (35). Diabetes is associated with a higher incidence of perioperative complications, such as the need for blood transfusions, pneumonia, delayed discharge, surgical site infections, and in-hospital mortality (36). Insomnia increased the risk of depression, inflammatory diseases, and infectious diseases, and led to an increase in all-cause mortality (37). Rigorous preoperative screening and intervention for comorbidities can help reduce surgical risks and alleviate patients’ psychological burden. Postoperative tracking revealed 1 case of wound infection and 1 case of dislocation at T3, and 5 cases of joint stiffness at T4. In addition to the effects of comorbidities, patients with postoperative complications showed lower PTG levels at T4. In addition to providing postoperative rehabilitation guidance, future research should continue to evaluate the effectiveness of continuous nursing interventions in reducing the incidence of complications.

At T1, patients with more OA-affected joints had lower PTG levels. The ongoing decline in joint function leads to decreased self-care ability, making patients more susceptible to negative psychological states such as anxiety and depression (6, 38, 39). In this study, joint function scores were positively correlated with PTG levels at all time points; better joint function was associated with higher PTG levels. However, at T2, TKA patients had lower PTG levels compared to THA patients. Previous studies have shown that pain resulting from joint function rehabilitation exercises is more pronounced in TKA patients compared to THA patients. As pain was a predictor of anxiety and depression, it contributed to lower levels of PTG in these patients (40–42). Personalized assessments led by physical therapists, integrating pain coping techniques with psychological strategies, are essential. For instance, dual-task training that combines physical exercise with cognitive engagement has been shown to break the conditioned response of pain during movement (43). Additionally, graded exposure therapy, which gradually expands patients’ functional activity thresholds, may significantly reduce the negative experiences associated with pain (44).

Coping styles play an important role in maintaining individuals’ psychological well-being and overall health. Positive coping strategies encourage individuals to adopt proactive approaches, thereby promoting both physical and psychological recovery (45, 46). Active coping behaviors are significantly positively correlated with rehabilitation adherence (30). However, patients undergoing THA/TKA often face various barriers to activity participation. For example, avoidance strategies may delay early mobilization and increase the risk of deep vein thrombosis, while submissive coping may lead to dependence on analgesics, hindering the recovery process (45, 47). In this study, preoperative medical coping scores consistently influenced PTG scores at various postoperative time points, suggesting the importance of assessing patients’ coping styles before surgery. Interventions should promote positive coping strategies, such as prehabilitation and cognitive behavioral therapy, which can involve progressive muscle relaxation and attentional redirection. Cognitive restructuring has been proven effective in reducing catastrophizing tendencies like pain-related fear and in building alternative, positive cognitions to better manage postoperative rehabilitation challenges (21, 48, 49).

General Self-Efficacy refers to an individual’s belief in coping with various stresses or challenges (50). Individuals with high self-efficacy not only set higher goals but also persistently strive to achieve them (51, 52). In our study, the trajectory of patients’ self-efficacy across four time points showed a positively correlated upward trend with PTG scores, with significant positive correlations at T1 and T2. This suggests that enhancing self-efficacy may be an important approach to promoting PTG and facilitating recovery. Strategies to improve self-efficacy may include developing structured educational programs that integrate preoperative and postoperative modular courses, covering pain management, rehabilitation training, and psychological adjustment (52, 53). Additionally, based on social cognitive theory, establishing a collaborative model between families and hospitals, such as involving family members in the development of rehabilitation plans, can further enhance patients’ self-efficacy (52).

### Limitations

This study has several limitations. First, the study participants were all from a single hospital where postoperative continuity services are provided, and all patients received standardized follow-up care. Therefore, the representativeness of the results may be limited. Future studies should include patients from hospitals of different levels and compare PTG levels between patients receiving continuity nursing services and those under traditional care models. Secondly, the data collection in this study was limited to 3 months post-surgery, a time when joint function generally stabilizes but has not yet reached the point where patients forget about the joint. Therefore, more long-term follow-up studies are needed to understand the patients’ PTG levels in the long term. Thirdly, although preoperative PTG is considered a baseline for growth related to chronic illness, the PTGI is inherently more suited to assessing responses to acute traumatic events. To address this, the present study employed hierarchical regression, controlling for joint function scores, to isolate the influence of psychological transformation mechanisms, such as coping strategies and self-efficacy, from physical recovery. Future studies should incorporate mixed methods (e.g., interviews) to triangulate and validate preoperative PTG experiences. Finally, we did not systematically collect chronic disease control indicators (e.g., HbA1c, blood pressure control rate), which may have led to an underestimation of the inhibitory effects of poorly controlled conditions on PTG. Future research should incorporate objective medical indicators to strengthen this analysis.

## Conclusion

PTG in hip and knee arthroplasty patients follows a dynamic trajectory—initial rise, decline at one month, and subsequent increase. The first postoperative month represents a key intervention window. Higher PTG is associated with better joint function, self-efficacy, positive coping, and social support, while avoidance and yield coping correlate negatively. As influencing factors vary over time, targeted education and interventions are needed to enhance PTG and support recovery.

## Data Availability

The raw data supporting the conclusions of this article will be made available by the authors, without undue reservation.
